# Taking pleasure seriously: Should alcohol research say more about fun? ‘No safe level’ advocates must take note

**DOI:** 10.1111/add.70050

**Published:** 2025-03-14

**Authors:** James Morris, Emma Davies

**Affiliations:** ^1^ London South Bank University – Psychology London UK; ^2^ Oxford Brookes University Oxford UK

**Keywords:** alcohol policy, alcohol risk messaging, behaviour change, drinking, public health, risk communication

*The pleasures of alcohol consumption are highly valued, particularly amongst people drinking above low‐risk levels. Improving risk awareness is important, but ‘no safe level’ and fear‐based messages may be ineffective or even counterproductive if they do not account for a complex range of social and cognitive moderators*.

Nicholls and Hunt propose three arguments for why pleasure should be taken more seriously in alcohol research and policy debates [1]. We concur with each, but focus here on the pragmatic case; namely, that ignoring the experienced pleasures of drinking can undermine public health efforts towards reducing alcohol harm.

Nicholls and Hunt highlight the limitations of health messages for behaviour change, notably observing that a public health ‘expert story’ of alcohol being inherently dangerous ‘runs hard against a public view of drinking’ as generally positive. We believe this is particularly important for public health actors to attend to, especially considering the increasing focus on cancer risks and ‘no safe level of consumption’ messaging. Psychological research has explored a range of phenomena that can inform public health messaging in this regard.

In addition to the risk of failing to change behaviour, messages that run against people's lived experiences risk backfire effects [2]. Even neutral alcohol risk messages have been found to evoke *defensive processing* amongst heavier drinkers, provoking denial, minimization or the avoidance of content, below conscious awareness [3]. This resistance appears to increase linearly with the level of consumption, indicating that the more individually relevant the risk information, the more likely that drinkers are to reject it [3]. This is not to infer that risk‐based messaging is redundant; rather, the mixed evidence on its effectiveness highlights the need for further understanding of the specific role of message content and key moderating processes, such as perceived relevance, emotional responses and self‐efficacy [3, 4, 5].

Qualitative research has also identified how heavier drinkers use personal exceptionalism and othering as strategies to construct and place their own drinking as ‘responsible’ in contrast to a stereotyped ‘other’ [6], including in response to alcohol health warnings [7]. Drinkers perceive a wide range of positives associated with drinking and are skilled at deploying these to resolve any dissonance invoked by incongruent information [6, 7, 8]. An evolving literature highlights how framing, stigma and identity processes may be involved in rejecting messages that are incongruent with a drinker's own experiences of alcohol use as being largely without problems and pleasurable [3, 4, 9, 10].

In addition to overlooking pleasure in drinking, ‘no safe level’ messaging may also reinforce the general perception that abstinence is the only acceptable goal. Indeed, abstinence can be perceived as threatening to drinkers and result in resistance or even the stigmatization of non‐drinkers [11]. Rather, it has been argued that an increasing recognition of moderation as an acceptable goal has a number of public health benefits [12]. A recent study identified that drinkers who had reduced their consumption had aimed to do so without ‘diluting the experience’ [13]. That heavier drinkers are both implicitly and explicitly resistant to ‘bad for health’ messages also accords with the value of motivational interviewing in treatment contexts. As heavy drinkers are well versed in the positives of their drinking, making space to examine both pros and cons in a non‐judgemental manner is necessary to facilitate change talk [14]. Relatedly, brief intervention approaches also avoid singularly framing drinking as ‘bad for health’, and instead offer normative feedback or gain‐based reasons to foster efficacy and motivation for change [15].

Effective messaging for alcohol behaviour change must therefore avoid identity threats and provoking excessive fear, and should ensure that the target outcomes are evaluated as being relevant and achievable [3, 4, 16]. Assessing how messages that reflect or omit pleasure impact the complex evaluations made by drinkers warrants exploration, particularly regarding the potential to increase relatability [4]. Problem recognition, the extent to which a drinker evaluates the level of risk/harm from their alcohol use with relative objectivity, is one variable that has been proposed for further development [5, 17]. Davies *et al*. (2024) reported *higher* drinking intentions when drinkers exposed to health risk information saw themselves as ‘responsible’ drinkers, highlighting the need to simultaneously address problem recognition if health risk messages are to work [4].

None of this is to say that improving knowledge of the risks of alcohol use is not important. Indeed, consumers have the right to know, and continued increases in alcohol‐related deaths requires urgent and multi‐component action. Increasing awareness also has the potential to increase policy support for alcohol public health policies [9]. Our argument is that public health actors must engage with the complex realities of behaviour change. A multidisciplinary approach, combining psychological insights and including the voices of people who drink ‘for pleasure’, will ensure that messaging has the greatest chance of reducing alcohol harm.

## AUTHOR CONTRIBUTIONS

**James Morris:** Conceptualization (lead); Writing—original draft (lead); writing—review & editing (equal). **Emma Davies:** Conceptualization (supporting); Writing—original draft (supporting); Writing—review & editing (supporting).

## DECLARATION OF INTERESTS

None.

## Data Availability

Not applicable.
